# Circulating Coding and Long Non-Coding RNAs as Potential Biomarkers of Idiopathic Pulmonary Fibrosis

**DOI:** 10.3390/ijms21228812

**Published:** 2020-11-20

**Authors:** Stefania Di Mauro, Alessandra Scamporrino, Mary Fruciano, Agnese Filippello, Evelina Fagone, Elisa Gili, Francesca Scionti, Giacomo Purrazzo, Antonino Di Pino, Roberto Scicali, Maria Teresa Di Martino, Roberta Malaguarnera, Lorenzo Malatino, Francesco Purrello, Carlo Vancheri, Salvatore Piro

**Affiliations:** 1 Department of Clinical and Experimental Medicine, Internal Medicine, Garibaldi-Nesima Hospital, University of Catania, 95122 Catania, Italy; 8stefaniadimauro6@gmail.com (S.D.M.); alessandraska@hotmail.com (A.S.); agnese.filippello@gmail.com (A.F.); giacomopurr@hotmail.it (G.P.); antonino.dipino@unict.it (A.D.P.); robertoscicali@gmail.com (R.S.); fpurrell@unict.it (F.P.); salvatore.piro@unict.it (S.P.); 2 Department of Clinical and Experimental Medicine, Respiratory Medicine Unit, A.O.U. “Policlinico-Vittorio Emanuele”, University of Catania, 95123 Catania, Italy; maryfruciano@yahoo.it (M.F.); efagone@unict.it (E.F.); elisagili@hotmail.com (E.G.); 3 Department of Experimental and Clinical Medicine, Magna Graecia University, 88100 Catanzaro, Italy; fscionti20@gmail.com (F.S.); teresadm@unicz.it (M.T.D.M.); 4 School of Human and Social Sciences, “Kore” University of Enna, 94100 Enna, Italy; roberta.malaguarnera@unikore.it; 5 Department of Clinical and Experimental Medicine, Unit of Internal Medicine, Azienda Ospedaliera Cannizzaro, University of Catania, 95100 Catania, Italy; malatino@unict.it

**Keywords:** RNAs, biomarkers, IPF, liquid-biopsy

## Abstract

Background: Idiopathic Pulmonary Fibrosis (IPF) is a chronic degenerative disease with a median survival of 2–5 years after diagnosis. Therefore, IPF patient identification represents an important and challenging clinical issue. Current research is still searching for novel reliable non-invasive biomarkers. Therefore, we explored the potential use of long non-coding RNAs (lncRNAs) and mRNAs as biomarkers for IPF. Methods: We first performed a whole transcriptome analysis using microarray (*n* = 14: 7 Control, 7 IPF), followed by the validation of selected transcripts through qPCRs in an independent cohort of 95 subjects (*n* = 95: 45 Control, 50 IPF). Diagnostic performance and transcript correlation with functional/clinical data were also analyzed. Results: 1059 differentially expressed transcripts were identified. We confirmed the downregulation of FOXF1 adjacent non-coding developmental regulatory RNA (FENDRR) lncRNA, hsa_circ_0001924 circularRNA, utrophin (UTRN) and Y-box binding protein 3 (YBX3) mRNAs. The two analyzed non-coding RNAs correlated with Forced Vital Capacity (FVC)% and Diffusing Capacity of the Lung for carbon monoxide (DLCO)% functional data, while coding RNAs correlated with smock exposure. All analyzed transcripts showed excellent performance in IPF identification with Area Under the Curve values above 0.87; the most outstanding one was YBX3: AUROC 0.944, CI 95% = 0.895–0.992, sensitivity = 90%, specificity = 88.9%, *p*-value = 1.02 × 10^−13^. Conclusions: This study has identified specific transcript signatures in IPF suggesting that validated transcripts and microarray data could be useful for the potential future identification of RNA molecules as non-invasive biomarkers for IPF.

## 1. Introduction

Idiopathic Pulmonary Fibrosis (IPF) is a progressive and lethal lung disease of unknown etiology, associated with a specific radiological and/or histological pattern of usual interstitial pneumonia. The disease is marked by a severe derangement of the lung architecture, causing cough and worsening dyspnea and ultimately leading to pulmonary failure and death. Several risk factors have been described contributing to IPF pathogenesis, although the real cause of the disease remains unknown [1]. According to the current pathogenetic hypothesis, recurrent microinjuries caused by chronic insulting agents, such as smoking or chronic gastroesophageal reflux, can stimulate an early macrophage response and chronic damage of alveolar epithelial cells, both responsible for the release of proinflammatory and profibrotic cytokines. These cytokines, inducing the activation of fibroblasts, may lead, in susceptible individuals, to an excessive and redundant reparative process that creates a progressive and irreversible fibrosis of the lung parenchyma [2,3].

IPF prognosis is poor; indeed, patients survive only 3–5 years from diagnosis. In addition, IPF clinical evolution is not predictable; indeed, it is slow and gradual in some cases or rapid and often dramatic in others.

Current treatments may slow down the progression of the disease, although the response to antifibrotic drugs may vary from patient to patient [1]. The diagnosis of IPF is complex and its clinical management suffers from the lack of reliable clinical non-invasive indicators. Many putative molecular markers, including proteins, cytokines or genetic and epigenetic variations have been studied in IPF and from time to time related to the correct diagnosis of IPF, severity of the disease, its prognosis and response to treatment [4]. Unfortunately, none of the studied biomarkers has demonstrated sufficient reliability and reproducibility to be regularly used in clinical practice. Among the wide spectrum of potential biomarkers, circulating RNAs are considered potential novel non-invasive diagnostic markers in different diseases [5,6,7]. Body fluid RNAs include noncoding small RNAs (e.g. microRNAs), known to negatively regulate gene expression through post-transcriptional mechanisms, and lncRNAs, which regulate genes at epigenetic, transcriptional, post-transcriptional, translational and post-translational levels [8].

Several lncRNAs have been reported to be dysregulated and involved in the regulation of pivotal molecular pathways in fibrosis diseases of the lung, kidney, liver, myocardium and peritoneum [9]. It has been reported that RNA molecules at the extracellular level are strongly resistant against endogenous RNase and various experimental conditions such as pH alterations, repeated freeze–thaw cycles, exogenous RNase treatments, extended Room Temperature (RT) incubation (24 h) and time delay in the processing of blood sample [10,11,12,13,14]. Furthermore, body fluid RNAs can be obtained through non-invasive ways and their levels can be easily detected through simple biology techniques such as Real-Time PCR [12].

In light of these considerations, the identification of dysregulated lncRNAs in body fluids such as serum or plasma may possibly help identify novel promising clinical diagnostic or prognostic biomarkers of fibrotic lung diseases and specifically of IPF. Therefore, the main aim of this study was to analyze the whole transcriptome profile of IPF subjects compared with controls, in order to identify potential novel non-invasive biomarkers able to simplify and improve IPF diagnosis, which still represents a clinical challenge.

## 2. Results

### 2.1. High Throughput Transcriptome Analysis

**Figure 1:**
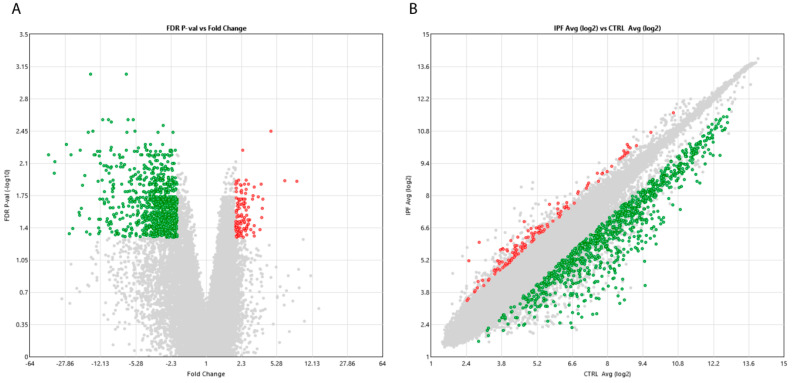
Volcano (**A**) and scatter plots (**B**) assessing the variation between Idiopathic Pulmonary Fibrosis (IPF) patients versus controls. The volcano plot visualizes the base 10 negative logarithm of the FDR-corrected *p*-values (Yaxis) and fold change deregulation values (X axis) (**A**). In the scatter plot, the values plotted on the X and Y axis are the averaged normalized signal values in each group (log_2_ scaled) (**B**). In both panels, red points in the plots indicate > 2.0-fold up-regulation of expression, green points indicate > 2.0-fold down-regulation of expression and gray points indicate < 2.0-fold change in expression. FDR-corrected *p*-value < 0.05.

In order to identify potential novel biomarker signatures associated with IPF, we performed a whole transcriptome analysis in sera from seven biopsy-proven IPF patients and seven healthy matched controls. Statistically significant differentially expressed transcripts in IPF patients compared with controls are represented as a volcano plot and scatter plot in Figure 1. We identified 1059 deregulated transcripts, among which 931 were up-regulated (Fold Change, FC > 2, False Discovery Rate, FDR-corrected *p*-value < 0.05) and 128 were down-regulated (FC < 2, FDR-corrected *p*-value < 0.05). Hierarchical clustering analysis of statistically significant deregulated transcripts showed distinguishable signatures in IPF patients with respect to the controls (Figure S1).

### 2.2. Differentially Expressed Transcripts are Associated with IPF Molecular Pathways

**Figure 2:**
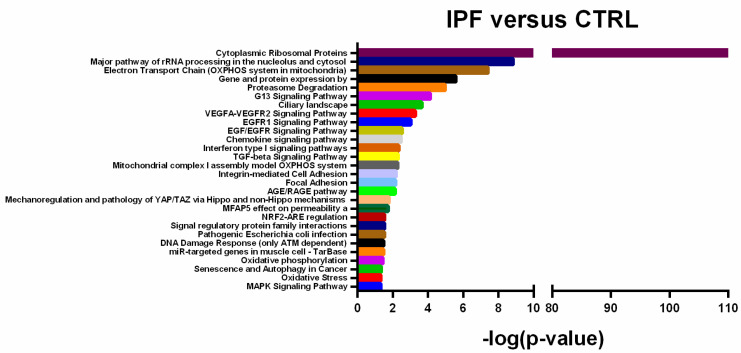
Enriched pathway analysis. Statistically significant pathways regulated by Differentially Expressed (DE) transcripts in Idiopathic Pulmonary Fibrosis (IPF) patients versus Control (CTRL). Fisher’s Exact Test: -Log (*p*-value) ≥ 1.13.

To gain insight into the potential biological functions of differentially expressed transcripts identified through microarray, we performed enriched biological pathway analysis through Transcriptome Analysis Console. Differentially Expressed (DE) transcripts were statistically significant (Fisher’s Exact Test *p* < 0.05), associated with several molecular pathways known to be involved in IPF pathogenesis, including reticulum endoplasmic stress and Unfolded Protein Response (UPR) pathways (“Cytoplasmic Ribosomal Proteins”, “Major pathway of rRNA processing in the nucleolus and cytosol”, “Proteasome Degradation”), mitochondrial stress and function (“Electron Transport Chain OXPHOS system in mitochondria”, “Oxidative Stress”), cell aging (“Senescence and Autophagy in Cancer”), cell adhesion (“Integrin-mediated Cell Adhesion”, “Focal Adhesion”), inflammatory pathways (“Chemokine signaling pathway”, “Gene and protein expression by JAK-STAT signaling after Interleukin-12 stimulation”, “Interferon type I signaling pathways”) cell proliferation and migration (“VEGFA-VEGFR2 Signaling Pathway”,”EGFR1 Signaling Pathway”, “EGF/EGFR Signaling Pathway”) and fibrogenesis pathways (“TGF-beta Signaling Pathway”) (Figure 2).

### 2.3. Validation of Deregulated Transcripts

Quantitative real-time PCR was used to confirm differential expression of four selected transcripts discovered by microarray analysis. Real time PCRs were performed in a larger independent cohort of 95 subjects (CTRL = 45, IPF = 50). Demographic characteristics (age, gender) and clinical data of the validation cohort subjects are reported in Table 1.

We selected the following transcripts: YBX3 and UTRN coding RNAs, FENDRR lncRNA and circ_0001924 circular RNA. We chose these transcripts because: (I) they presented a high level of fluorescence microarray signals (averaged normalized log_2_ scaled signal values ≥5); (II) they presented an FDR *p*-value ≤ 0.02; (III) they had at least a five FC deregulations.

**Figure 3:**
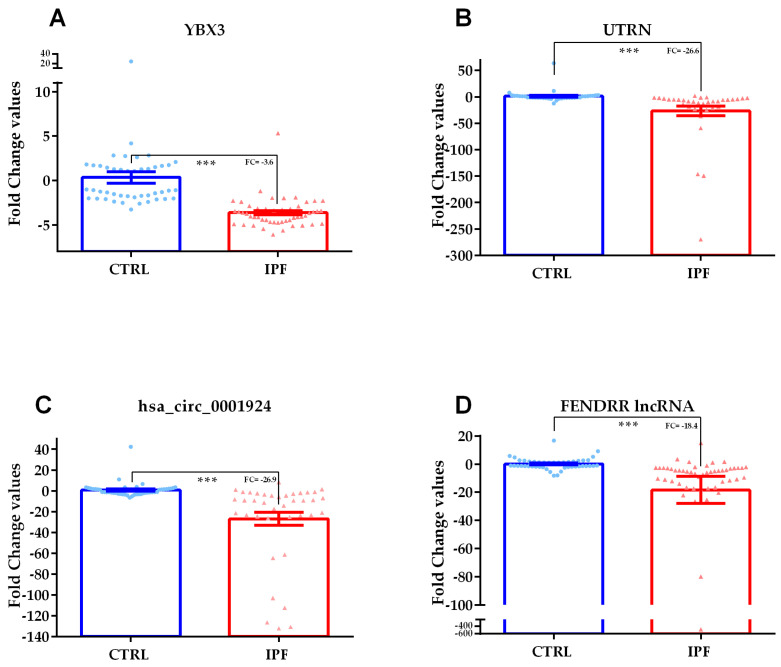
Scatter plots with bars of YBX3 (panel **A**) and UTRN coding RNAs (panel **B**), hsa_circ_0001924 circular RNA (panel **C**) and FENDRR long non-coding RNAs (lncRNA) (Panel **D**) validated through qPCR in serum samples of IPF patients versus CTRL. The Mann–Whitney U test was used for YBX3 coding RNA that presented a non-parametric distribution, while un-paired t-test was used for all the other transcripts showing parametric distributions. *n* = 95: 45 CTRL, IPF 50. *** = *p*-value ≤ 10 × 10^−10^. FC = Fold Change.

Accordingly, to microarray data, analyzed transcripts had a decreasing expression trend in IPF versus CTRL comparison; all analyzed transcripts had a very strong statistical significance: YBX3 FC = −3.6 *p*-value = 3.5 × 10^−14^, UTRN FC = −26.6 *p*-value = 1.99 × 10^−10^, FENDRR FC = −18.4 *p*-value = 5.18 × 10^-10^, circ_0001924 FC = −26.9 *p*-value = 2.67 × 10^−11^. Transcript expression values in IPF patients versus CTRL are reported as scatter plots with bars in Figure 3.

### 2.4. Validated Transcripts are Associated with Lung Function Parameters

We performed a correlation analysis to test the hypothesis of a potential correlation between analyzed transcript expression values and pulmonary functional parameters, in particular FVC% and DLCO%, both commonly measured during spirometry tests and predictors of disease severity.

We computed the Pearson correlation for FVC% and the Spearman correlation for DLCO% since they had a parametric and non-parametric distribution, respectively.

FVC% data showed a positive relationship with FENDRR lncRNA and hsa_circ_0001924 circRNA expression values. Therefore, when these ncRNA expression levels decrease also FVC% values fall. Both transcripts were also significantly associated through the Spearman correlation with DLCO% values. We also analyzed if all validated transcripts correlated with the “pack years” index, which reflects cigarette smoke exposure. Only YBX3 and UTRN coding RNAs were associated though the Spearman correlation to this index. Table 2 shows *p*-values and R^2^ values of the correlation analysis.

### 2.5. Receiver Operating Characteristic Curve Analysis Shows High Transcript Diagnostic Performance

**Figure 4:**
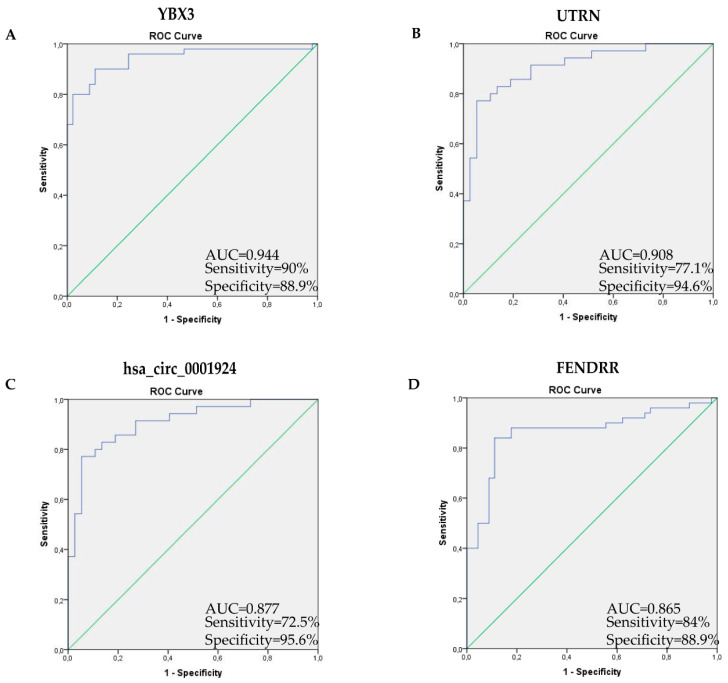
Receiver Operating Characteristic (ROC) curve analysis for predicting YBX3, UTRN, hsa_circ_0001924 and FENDR as IPF patient diagnostic biomarkers with respect to the controls. YBX3 AUC = 0.944 (95%CI = 0.895–0.992 sensitivity = 90%, specificity = 88.9%, *p* = 1.02 × 10^−13^) (**A**); UTRN AUC = 0.908 (95% CI = 0.840–0.976 sensitivity = 77.1%, specificity = 94.6%, *p* = 2.61 × 10^−9^) (**B**); hsa_circ_0001924 AUC = 0.877 (95% CI = 0.800–0.954, sensitivity = 72.5%, specificity = 95.6%, *p* = 2.38 × 10^−9^) (**C**); FEDRR AUC = 0.865 (95%, CI = 0.787–0.943 sensitivity = 84%, specificity = 88.9%, *p* = 9.39 × 10^−10^) (**D**).

The diagnostic power of validated transcripts for IPF patient identification was analyzed through Receiver Operating Characteristic (ROC) curve analysis. All analyzed transcripts showed an excellent diagnostic performance. The most powerful transcripts were YBX3 and UTRN coding RNAs: YBX3 (Area Under the Receiver Operating Characteristic AUROC 0.944, CI 95% = 0.895–0.992, sensitivity = 90%, specificity = 88.9%, *p*-value = 1.02 × 10^−13^); UTRN (AUROC 0.908, CI 95% = 0.840–0.976, sensitivity = 77.1%, specificity = 94.6%, *p*-value = 2.61 × 10^−9^). Moreover, hsa_circ_0001924 and FENDRR lncRNAs had a high diagnostic performance: hsa_circ_0001924 (AUROC 0.877, CI 95% = 0.800–0.954, sensitivity = 72.5%, specificity = 95.6%, *p*-value = 2.380 × 10^−9^); FENDRR (AUROC 0.865, CI 95% = 0.787–0.943, sensitivity = 84%, specificity = 88.9%, *p*-value = 9.39 × 10^−10^) (Figure 4). *p*-values in bold are statistically significant.

## 3. Discussion

IPF is a lung disease characterized by a relentless and progressive fibrosis of lung parenchyma. Its etiology is unknown and its pathogenesis not yet completely understood. In spite of the current antifibrotic drugs, which may slow down the progression of the disease, the prognosis of IPF remains poor. In IPF, symptoms, such as cough and dyspnea, are non-specific, therefore making diagnosis and staging of the disease particularly difficult, especially in those patients lacking the typical radiological Usual Interstitial Pneumonia (UIP) pattern at High-Resolution Computed Tomography (HRCT). In these patients, who represent more than 50% of cases, lung biopsy may represent a diagnostic solution, although not always indicated because of advanced age and poor lung function of most IPF patients. In addition, lung biopsy may induce acute exacerbations of the disease in some cases. All these diagnostic limitations make the need for an earlier, non-invasive and confident compelling diagnosis biomarker [4]. Several biomarkers have been investigated as potentially useful elements in the diagnosis, staging and prognosis of IPF. Unfortunately, none of the molecular biomarkers studied so far have been shown to be clinically relevant. Circulating non-coding RNAs have recently received considerable interest. In a study published in 2015, Yang et al. showed, for the first time, a substantial difference in the expression of miRNA 47 in the serum of IPF patients compared with healthy controls [15], suggesting a pathogenic role for miRNAs in IPF and paving the way for the study of RNAs as potential non-invasive biomarkers for IPF. Most of the subsequent ncRNA-related biomarker research in lung diseases has been focused on miRNAs. However, no single miRNA has been found to be analytically validated and clinically useful as a biomarker in this research field. This study supports the idea that other classes of RNA molecules, especially mRNAs and lncRNAs, could serve as useful biomarkers in diseases such as IPF. The simplicity in their dosage and their stability in biological fluids make these RNAs perfect candidates for this purpose. Accordingly, one of the main aims of this study was to analyze the whole transcriptome, in particular lncRNAs and serum mRNAs expressed in patients with IPF, in order to identify “molecular fingerprints” useful for a more accurate definition of IPF diagnosis and prognosis. For this, we used microarray analysis to perform an initial screening of the whole transcriptome expressed in the serum of IPF patients in comparison with healthy controls (*n* = 14: 7 IPF, 7 CTRL). We identified 1059 deregulated transcripts; 128 up-regulated and 931 down-regulated (FDR-corrected *p*-value < 0.05). Interestingly, computational analysis showed that these transcripts were associated with pathways consistent with IPF pathogenesis, including fibrogenesis, inflammation, senescence, adhesion, proliferation and migration.

Based on the level of deregulation and statistical FDR *p*-values four different transcripts including non-coding RNAs (FENDRR lncRNA and hsa _circ_0001924 circular RNA) and coding RNAs (YBX3 and UTRN) were selected for subsequent qPCR validation. They all were found to be downregulated in IPF patients compared with controls (*n* = 95: CTRL = 45, IPF = 50), with a very strong statistical significance (*p*-values in the range of E−10).

These sets of RNAs may constitute new non-invasive biomarkers for the IPF pathophysiological state; indeed, in our study, the four analyzed transcripts had considerable diagnostic power for the discrimination of IPF patients with respect to the controls. Consistently, the relative ROC curves showed remarkably encouraging results for all the four analyzed transcripts with AUC values ranging from 0.865 to 0.944, sensitivity values varying from 72.5% to 90% and specificity values ranging from 88.9% to 95.6%.

In addition to transcript outstanding diagnostic performances, the significant correlation between the expression levels of FENDRR and hsa_circ_0001924 with the respiratory functional parameters FVC% and DLCO% should be highlighted, suggesting their potential prognostic value, though their deregulation will be have to be validated in longitudinal cohorts. Cigarette “pack-years”, a well-known IPF risk factor, was instead correlated with the expression levels of YBX3 and UTRN.

Moreover, it is important to report that a direct association between FENDRR lncRNA expression and IPF pathogenesis was observed in previous studies. In more detail, it has been demonstrated that FENDRR is downregulated in IPF, and its downregulation contributes to increased fibroblast activity and pulmonary fibrosis [16,17]. As far as hsa_circ_0001924 is concerned, there are no studies that evaluate its dysregulation or involvement in any kind of disease; therefore, it could be interesting to analyze its unexplored functional role in further studies. In the end, nevertheless, YBX3 and UTRN are not directly associated with IPF, it has been reported the involvement of these coding RNAs in claudin-mediated cell proliferation regulation, which is impaired in lung diseases [18,19] and Duchenne Muscular Dystrophy (DMD) fibrosis, respectively [20,21].

Our data could provide the basis for the identification of lncRNAs and mRNAs as classes of biomarkers allowing for an early diagnosis of IPF and contributing to improve the diagnostic accuracy of clinical data combined with imaging. However, these data should be confirmed in a larger independent external cohort. Serum RNAs should be studied in animal models in vivo, so that factors able to influence transcriptome, such as genetic and environmental factors, could be minimized. The prognostic utility of the identified markers should be evaluated through longitudinal studies, comparing the difference of expression of these molecules in a serial manner and in relation to the variation of other functional and non-functional parameters, in order to establish whether they can be useful as staging markers for IPF, alone or in association with other variables currently considered in clinical practice. The longitudinal study should also evaluate the expression of biomarkers before and after treatment with antifibrotics to evaluate the response to drugs as part of treatment management. Finally, it could be interesting to analyze whether observed transcript serum deregulation is also reflected in biopsies of IPF patients and in cellular models to establish a potential involvement of these transcripts in IPF pathogenetic pathways. This involvement should also be validated by functional experiments of transient induction or inhibition in vitro. Even with these limitations, this study offers a new and promising approach to the urgent need for biological markers able to make IPF clinical management easier, as well as paving the way for emerging concepts implementing the understanding of IPF pathophysiology.

## 4. Materials and Methods

### 4.1. Study Subjects

The study cohort included 52 healthy controls and 57 patients with IPF, the first were enrolled from the Internal Medicine Unit of the Garibaldi-Nesima Hospital, University of Catania, Italy and the second were recruited in the Centre for Interstitial and Rare Lung Diseases at the University of Catania, Italy. In accordance with the American Thoracic Society/European Respiratory Society ATS/ERS criteria for the diagnosis of IPF, clinical, physiologic, and high-resolution computed tomography data of these patients supported the diagnosis of IPF. Blood samples were collected from IPF patients at the time of diagnosis and retrospectively grouped.

The whole cohort included a discovering cohort of 7 healthy controls and 7 IPF patients and a validation cohort of 45 healthy controls and 50 IPF patients; in both cases, controls were matched for age, gender and cigarette smoke exposure.

Anthropometric indexes were collected for all the study participants; respiratory function data (FVC%, DLCO%) were collected for IPF patients. Individuals affected by cancer, rheumatologic diseases or other lung diseases were excluded from our study.

All subjects gave their informed consent for inclusion before they participated in the study. The study was conducted in accordance with the Declaration of Helsinki, and the protocol was approved by the Ethics Committee of ethics committee Catania 2 (702/CE, SMP-01, 23 Sep 2016, n.28/2016/CECT2).

### 4.2. Sample Processing

Blood samples were collected in Vacutainer (Vaud, Switzerland) tubes and serum was isolated from whole blood through centrifugation at 1500× *g* for 10 min at RT. The upper-layer supernatant was collected and aliquots were stored at −80 until analysis.

### 4.3. RNA Isolation

Total RNA was isolated from 400µL of serum by using the miRNeasy mini kit (Qiagen, Hilden, Germay)according to the manufacturer’s instructions. RNA concentration and quantification were performed as previously reported [22,23].

### 4.4. Microarray Analysis

The high-throughput profiling of coding/non-coding RNAs in serum samples of seven biopsy-proven IPF patients and seven matched healthy controls was performed by using Clariom D Pico Assay (Thermo Fisher Scientific, Milan, Italy) technology according to the manufacturer’s instructions [7].

### 4.5. Computational Analysis

To elucidate the function of statistically significant deregulated transcripts in IPF patients versus controls, pathway enrichment analysis was performed using Transcriptome Analysis Console v.4 (Thermo Fisher Scientific, Santa Clara, CA, USA), which can extrapolate canonical biological pathways from the WikiPathways (Thermo Fisher Scientific, Santa Clara, CA, USA) database and to return *p*-values applying a two-sided Fisher’s Exact Test (−log *p*-value ≥ 1.03).

### 4.6. Single Real-Time PCR Assays

In order to validate microarray results, the expression of four selected transcripts was analyzed by single real-time PCR assays in serum samples of the validation cohort (*n* = 95: 50 IPF, 45 CTRL). qPCR analysis was performed by using the Power SYBR Green RNA-to-CT1-Step Kit (catalogue number: 4389986, Thermo Fisher Scientific, Milan, Italy) in QuantStudio 5 Real-time PCR System (Thermo Fisher Scientific, Singapore, Singapore). Through this kit, in a single reaction we performed both RTs and qPCRs using 20 ng of total RNA as reaction input and according to the following thermal cycle: 48 °C for 30 min (RT), 95 °C for 10 min (Activation of AmpliTaq Gold DNA Polymerase), followed by 40 cycles of 95 °C for 15 s (denaturation) and 60 °C for 1 min (annealing/extension). Expression fold changes were calculated by applying the 2^−ΔΔCt^ method, using GAPDH as a reference gene and healthy control subjects as a calibrator group [24]. PCR primers were designed by using Primer Blast (https://www.ncbi.nlm.nih.gov/tools/primer-blast/). For circular RNA, we used divergent primers complementary to the back-splicing junction identified through CircInteractome (https://circinteractome.nia.nih.gov/). Primer sequences are reported in Table 3.

### 4.7. Statistical Analysis

Microarray data normalization and DE coding and non-coding RNA identification were performed by using *Transcriptome* Analysis Console (TAC) Software v.4 (Thermo Fisher Scientific, Santa Clara, CA, USA) according to the following parameters: Analysis Type: Expression Gene, Summarization Method: Gene Level—RMA, FDR-corrected *p*-value < 0.05.

In order to evaluate whether clinical and expression data were normally or not normally distributed, we applied three tests: D’Agostino-Pearson omnibus test, Shapiro–Wilk normality test and Kolmogorov–Smirnov normality test. Data were considered normally distributed if the results were concordant for all three tests. Unpaired t-test or Mann–Whitney U-test was used to analyze the statistical significance of real-time expression results for parametric or no parametric data, respectively.

We performed linear regression analysis or Spearman correlation (respectively, for normally or not normally distributed data) in order to identify a potential relationship between transcript expression values and subject functional/clinical data. The statistical analyses were performed by using GraphPad prism 6.0 (GraphPad Software, Inc., San Diego, CA, USA).

ROC curves and AUC were used to assess the diagnostic performance of identified differentially expressed RNAs for discrimination of IPF patients with respect to the controls. SPSS PASW Statistics was used for ROC curve analysis [7].

## Figures and Tables

**Table 1:** Demographic and clinical data of the validation cohort. T-test or Mann–Whitney U-test were applied to check the statistical significance of parametric and nonparametric demographic and clinical data, respectively. Categorical variables were assessed through chi-square test. Data are presented as means ± standard deviation for normally distributed variables, as percentages for categorical variables, or as median ± standard deviation for non-normal variables. BMI: Body Mass Index, FVC: Forced Vital Capacity, DLCO: Diffusing Capacity of the Lung for carbon monoxide.

Clinical Characteristics	Control (*n* = 45)	IPF (*n* = 50)	*p*-Value
Gender (% M)	60	78	0.216
Age (years)	68.24 ± 8.53	71.32 ± 8.28	0.078
Weight (Kg)	74.59 ± 14.98	73.12 ± 13.25	0.617
Height (cm)	167 ± 9.19	164 ± 9.79	0.131
BMI (kg/m^2^)	27.06 ± 4.02	27.56 ± 3.54	0.525
Pack-years	15 ± 25.70	20 ± 33.83	0.790
FVC%		82.02 ± 19.08	
DLCO%		55 ± 14.76	

**Table 2:** *p*-values and eventual R^2^ values of correlation analysis between coding/non-coding RNAs and clinical data. Pearson correlation was applied for continuous variables while Spearman correlation was applied if one of the two sets of variables was not normally distributed. Statistical significance was established at *p* < 0.05.

**PEARSON CORRELATION**		
	**FVC %**	
	*p*-value	R^2^
FENDRR	**0.035**	0.07
hsa_circ_0001924	**0.033**	0.09
**SPEARMAN CORRELATION**		
	**DLCO %**	**PACK YEARS**
	*p*-value	*p*-value
FENDRR	**0.008**	0.139
hsa_circ_0001924	**0.016**	0.454
UTRN	0.228	**0.038**
YBX3	0.388	**0.034**

**Table 3:** PCR primer sequences. PCR primers were designed for selected transcripts and GAPDH housekeeping gene.

	Forward	Reverse
hsa_circ_0001924	ACCATGGCGAGTGGAAGAAAT	TTTAATCTAGGTGAAATGCCCCTT
FENDRR	CACGAAAGGTGGTGCCGAGA	TCCGTTTGCATCCAACATTGTCT
UTRN	CCTGACAATGGGCAGAACGA	CCCACTCTTTGAAAATCGAGCA
YBX3	AGCGAAGACGCGGAGAAAAA	TCCATTTCTGACGTTGAACCA
GAPDH	TGCACCACCAACTGCTTAGC	GGCATGGACTGTGGTCATGAG

## Supplementary Materials

The following are available online at https://www.mdpi.com/1422-0067/21/22/8812/s1, Figure S1: Hierarchical clustering of DE transcripts between IPF patients versus Controls.

## Abbreviations

IPF	Idiopathic Pulmonary Fibrosis
lncRNA	Long Non-Coding RNA
FENDRR	FOXF1 adjacent non-coding developmental regulatory RNA
UTRN	utrophin
YBX3	Y-box binding protein 3
FVC	Forced Vital Capacity
DLCO	Diffusing Capacity of the Lung for carbon monoxide
AUC	Area Under the Curve
RT	Room Temperature
FC	Fold Change
FDR	False Discovery Rate
DE	Differentially Expressed
UPR	Unfolded Protein Response
BMI	Body Mass Index
FVC	Forced Vital Capacity
DLCO	Diffusing Capacity of the Lung for carbon monoxide
ROC	Receiver Operating Characteristic
AUROC	Area Under the Receiver Operating Characteristics
UIP	Usual Interstitial Pneumonia
HRCT	High-Resolution Computed Tomography
DMD	Duchenne Muscular Dystrophy
