# Supplementary material for: Circulating Coding and Long Non-Coding RNAs as Potential Biomarkers of Idiopathic Pulmonary Fibrosis
Source: Int J Mol Sci. 2020 Nov 20;21(22):8812. doi: 10.3390/ijms21228812 (PMC7709007; doi:10.3390/ijms21228812)
Supplement: Supplementary file 1 [file ijms-21-08812-s001.zip › Supplementary Material.pdf]

SUPPLEMENTARY DATA

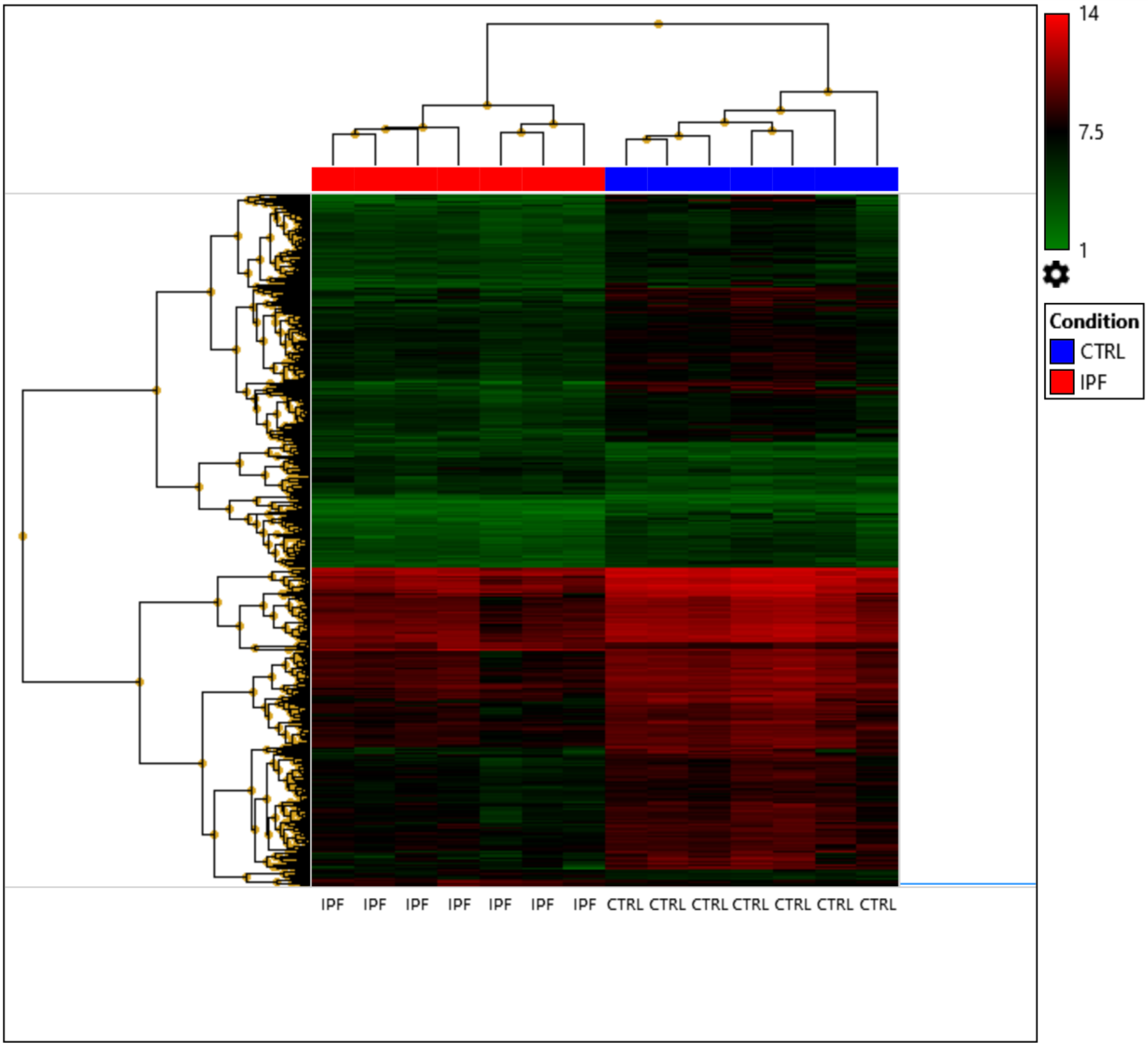

Supplementary figure 1: Hierarchical clustering of DE transcripts between IPF patients versus Controls.

Each row represents the relative expression level of a single transcript and each column represents a single analyzed sample. Fold change >2.0 or <-2 and FDR-corrected p-value<0.05.
